# Cyberbullying Involvement and Psychological Distress among Chinese Adolescents: The Moderating Effects of Family Cohesion and School Cohesion

**DOI:** 10.3390/ijerph17238938

**Published:** 2020-12-01

**Authors:** Xi Zhang, Ziqiang Han, Zhanlong Ba

**Affiliations:** 1 Natural Language Processing Group, Nanjing University, Nanjing 210023, China; xavier_ar7@hotmail.com; 2 School of Political Science and Public Administration, Shandong University, Qingdao 266237, China; ziqiang.han@sdu.edu.cn; 3 School of Sociology, Beijing Normal University, Beijing 100875, China

**Keywords:** cyberbullying, China, psychological distress, child, adolescent, family cohesion, school cohesion, moderating

## Abstract

Cyberbullying and its consequences is a little-investigated public health issue. We investigated the correlations between cyberbullying involvement, either being a victim or being a preparator, and psychological distress among a group of Chinese adolescents. A representative sample of 4978 students from Jiangsu province covering all types of pre-college schools was surveyed using a stratified sampling method. Both being a victim and being a perpetrator correlated with higher degrees of psychological distress, and the former’s effect is stronger. Family cohesion and school cohesion are protective factors of psychological distress, but only family cohesion plays a moderating effect between cyberbullying involvement and distress. Moreover, the positive correlations between cyberbullying involvement and psychological distress become non-significant when the interactions are included in regression models. Last but not least, female students and students in a higher grade or students with worse academic performance have higher degrees of distress. Our study reveals that, instead of school cohesion, family cohesion is more important to mitigate the psychological impact of cyberbullying involvement and eventually heal the trauma.

## 1. Introduction

Both the nice and the ugly aspects of humanity, such as bullying and violence in the physical world, can emerge and migrate to cyberspace with the increase of human activities in cyberspace and the integration of the physical and virtual world. Generation Z, born after the late 1990s, are growing up as the Internet blossoms, and activities in cyberspace have become an unneglectable component of their daily life. The percentage of youth connected to the Internet in the United States was 97% ten years ago, and has now reached a much higher share [1]. The co-occurrence of human behavior in physical space and cyberspace is more prevalent in society, such as China, which is open to new technologies and the adoption of information technology and application in particular. With the fast development of smartphones, tablets, and smartphone applications and the integration of online-offline services in the last decade, more and more people have become dependent on the Internet and smartphones. According to the 46th China Statistical Report on Internet Development, there are 940 million netizens in China in June 2020, sharing 67.0% of the total population [2]. Within them, 99.2% use smartphones to access the Internet. Another report about children and adolescents’ adoption of the Internet showed that 99.2% of the Chinese children and adolescents have experience of accessing the Internet in 2020, and 78% of the youth started to use internet service under the age of 10 [3]. The vast and deep integration of virtual and physical life, such as online shopping, digital payment, online gaming, online socialization, online study, etc., has made human activities in cyberspace much more prevalent than ever, and this trend will keep continuing in the predictable future.

Cyberbullying is becoming more common with children and adolescents’ increasing activities in cyberspace [4]. It refers to acts intended to harm others who cannot defend themselves using information communication technologies (ICTs) or in cyberspace [5,6]. Generally, there are two types of cyberbullying assessment instruments [7,8,9]. The first type usually describes or defines cyberbullying first and then asks the respondents to rate the prevalence or frequency of cyberbullying involvement. The other kind usually inquires about the occurrence of concrete cyberbullying behaviors, such as verbal or relational bullying in cyberspace. The prevalence of cyberbullying perpetration, victimization, and their overlap varies in different countries and research contexts, and there has been an increasing trend in recent years [10]. A recent review found that Canada and China have a higher prevalence of cyberbullying from the victim’s perspective, while Australia, Sweden, and Germany have a relatively lower occurrence rate compared with other countries included in the survey. The median prevalences in these countries from the studies included in this review were 23.8% (Canada), 23.0% (China), 5.0% (Australia), 5.2% (Sweden), and 6.3% (Germany) [10], while the prevalence of being a perpetrator, a victim or both in the United States ranged from 1% to 41%, 3% to 72%, and 2.3% to 16.7%, respectively [11]. Cultural differences and Internet accessibility are the possible reasons for such a difference across these countries [10]. This review also showed a lack of cyberbullying studies from China, a similar observation from another school bullying review in 2010 [12]. Thus, more studies on cyberbullying in China should be conducted.

Cyberbullying experience can cause various mental health, social-psychological, and behavioral problems, such as emotional and psychological distress, social anxiety [13], aggression and hostility [14], fear, depression, hyperactivity disorder, substance use [13], self-harm [15], and even suicidal ideation and attempts [13,14,15,16]. As the early stage of the consequence of cyberbullying involvement, psychological distress can lead to other severe mental health and behavior problems, such as suicide [17]. Prior studies indicated that cyberbullying’s indirect effect on suicidal ideation through psychological adjustment was actually more massive than cyberbullying’s direct effect [18]. Moreover, a study from Canada indicated that the cyberbullying victimization experience predicted psychological distress and low self-esteem over other bullying forms in schools [19]. Meanwhile, cyberbullying perpetration is not only correlated with psychological difficulties and lower quality of life [20] but is also associated with external problematic behaviors over time [21]. Therefore, it is essential to investigate the impact mechanisms between cyberbullying involvement and psychological distress and protective factors.

There are generally two groups of moderators between cyberbullying experience and the social-psychological consequences: the contextual factors, such as school climate, family relationship, and individual factors, such as gender, age, and sexual orientation [14]. Investigating the contextual factors is crucial because they are changeable and can provide scientific lessons and solutions for further interventions. A prior study indicated that social support from family, teachers, and friends could reduce the negative psychosocial symptoms while increasing the well-being of cyberbullying-involved students [22]. Moreover, school and family are the two micro-social environments that students interacted with in daily life, and, thus, we included the school and family factors as the moderating factors in this analysis with a firm purpose of intervention in the future. 

Based on the reviews about the prevalence of cyberbullying, the associations of cyberbullying involvement and the social-psychological consequences, and the roles of family cohesion and school cohesion, we found a knowledge gap in the correlations between cyberbullying involvement and its psychological consequences. Therefore, we analyzed a recently collected representative database from Jiangsu province in China with the purpose of investigating the correlations between cyberbullying involvement and one of the most common mental health issues—psychological distress—with a focus on the mitigating effects of family cohesion and school cohesion. We assumed that:

**Hypothesis 1a** **(H1a).** Being a cyberbullying victim is correlated with a higher degree of psychological distress.

**Hypothesis 1b** **(H1b).** Being a cyberbullying perpetrator is also correlated with a higher degree of psychological distress.

**Hypothesis 2a** **(H2a).** Family cohesion can mitigate the impact of cyberbullying victimization on psychological distress.

**Hypothesis 2b** **(H2b).** Family cohesion can mitigate the impact of cyberbullying perpetration on psychological distress.

**Hypothesis 3a** **(H3a).** School cohesion can reduce the impact of cyberbullying victimization on psychological distress.

**Hypothesis 3b** **(H3b).** School cohesion can reduce the impact of cyberbullying perpetration on psychological distress.

## 2. Methods 

### 2.1. Participants and Sampling 

Four thousand nine hundred seventy-eight (4978) adolescents from Jiangsu province, China, participated in this study between December 2019 and January 2020. A stratified sampling method was adopted in this survey. We first divided Jiangsu providence geographically into the north, central and south parts. The geographical variance also represents the economic differences within the province. One rural county and one urban county/district were randomly selected within each of the three geographical areas. One primary school, one middle school, and one high school were randomly selected within each county. Only students from grades four to six within the primary schools participated in the survey because we assumed that the youngers might be too young to understand our questions. Thus, children from grade four to grade six in primary schools, students of all grades in middle school and high school participated in the survey, representing the adolescents within Jiangsu province.

The survey was conducted through computer-assisted solutions. We developed and distributed our questionnaire through an online survey platform, WJX (www.wjx.cn). Working closely with the local education administrative agencies and the schools, a link to our online questionnaire survey was distributed to the qualified candidates. The students can finish the self-reported survey either through a computer or smartphone, or electronic tablets. Students from the selected schools finished the survey using computers or tablets during their IT (information and technology) class in most of the participated schools. The first page of the questionnaire was displayed, and a student can finish it voluntarily and anonymously, and they can quit the survey at any time if they do not like it. The ethical research committee of Sichuan University approved the IRB (K2019067, 12 November 2019) for all data collection efforts targeting both parents, teachers, and students, and we only used the children’s data in this paper.

### 2.2. Measures

Psychological distress: The psychological distress scale from the Psychological Well-Being and Distress Screener (PWDS) [23] was used to measure adolescents’ mental health status. The PWDS is a 10-item scale consisting of two five-item subscales, namely the psychological well-being scale and psychological distress scale. The PWDS was developed from prior measures of adolescents’ mental health status in a large cross-countries study, the Health Behavior in School-Aged Children (HBSC), organized by the World Health Organization (WHO). The PWDS was initially tested in the United States by a sample of youth in Grades 5 to 10 [23] and later expanded to other cultures, such as the Chinese [24]. The psychosocial distress scale consists of five items, inquiring about a respondent’s feelings of psychological distress. The inquiries were, “Thinking about your last week, have you felt…?” “How often have you had the following…”. “sad” and “lonely” were the emotions for the last week, while “feeling low,” “feeling nervous,” and “feeling irritability or bad temper” was the feelings for the last six months. The answers evaluating each of the feelings were “Never (1)”, “Rarely (2)”, “Sometimes (3)”, “Frequently (4),” and “Always (5)”. The psychological distress scale’s five items exhibited good measurement qualities and internal consistency with a Cronbach alpha test result of 0.760 in a Chinese sample from Hebei province [24]. In our study, the Cronbach alpha result was 0.8987, indicating very good internal consistency. The aggregation of the five items’ scores was used as the psychological distress degree, ranging from 5 to 25, with a mean value of 11 and a standard deviation of 4.35 (Table 1).

The cyberbullying investigated the students’ involvement in four types of bullying behaviors in cyberspace, based on measures used in the School Crime Supplement (SCS) of the National Crime Victimization Survey [25] developed by the United States’ National Center for Education Statistics and in our prior studies [26,27,28,29]. The first one was, “insulating or laughing or mocking others through a mobile phone or in cyberspace, including the Wechat, Tik Tok, Tencent QQ, Weibo, or other social media platforms or apps.” The second behavior was “spreading rumors or negative information about others,” while the third one was “violating other’s privacy concerns by posting other’s personal information, photos or videos online.” The last one was “Excluding or isolating others in online games or other social interactions in cyberspace.” The cyberbullying victimization was obtained by the question “Have any of your classmates or peers engaged in the following behaviors to you in last year?” while the cyberbullying perpetration question was “have you engaged in the following behaviors to your peers or classmates in last year?”. The original answers to each of the four behaviors described were “never,” “rarely,” “sometimes,” and “often,” and the “sometimes” and “often” choices were coded as one (involved in cyberbullying) while the “never” and “rarely” choices were coded as zero (not involved in cyberbullying) due to the repetitiveness definition of bullying [30]. If one respondent experienced any of the four cyberbullying behaviors proposed, he or she was defined as being a cyberbullying victim or cyberbullying perpetrator. Within the sample, 7.49% of the students were self-reported cyberbullying victims, while 2.05% were self-reported cyberbullying perpetrators (Table 1).

The school cohesion was measured by two items, while three items captured the family cohesion. All items were measured by five-point Likert scales. The two questions about school cohesions were “how is your relationship with your teacher?” and “how is your relationship with your classmates?” and the answers ranged from one to five, representing the meanings from “very bad (1)” to “very good (5)”. The three statements about family cohesion were “how is your relationship with your parents?”, “do you like to share your thoughts and feelings with your parents?” and “do you like to stay with your family?”. The sum of the items within each category was used to score the school cohesion and family cohesion. The school cohesion score ranged from 2 to 10, with a mean value of 8.46 and a standard deviation of 1.41, while the family cohesion degree was 12.82 on average, ranging from 3 to 15 (Table 1). The Cronbach’s alpha test results of the family cohesion indicator and the school cohesion indicator were 0.8352 and 0.7789, respectively, indicating good internal consistency of both measures.

Gender, grade, whether being in a boarding school, and academic performance were included as control variables. Within the 4,978 students, 51.45% of them were boys, 23.89% of them living in school during weekdays, and with an average self-evaluated academic performance of 3.40 ranging from one to five (Table 1).

### 2.3. Analysis Strategy 

We firstly reported the descriptive analysis results in Table 1. The correlations between the cyberbullying involvement (being a victim and being a perpetrator) and the psychological distress were analyzed using Ordinary Least Squares (OLS) regressions. Both the adjusted and unadjusted results were reported. The moderating effects of family cohesion and school cohesion were tested in the models using interactive variables. All analyses were conducted by the statistical software Stata 15.1 (StataCorp LLC, College Station, TX, USA).

## 3. Results

**Figure 1:**
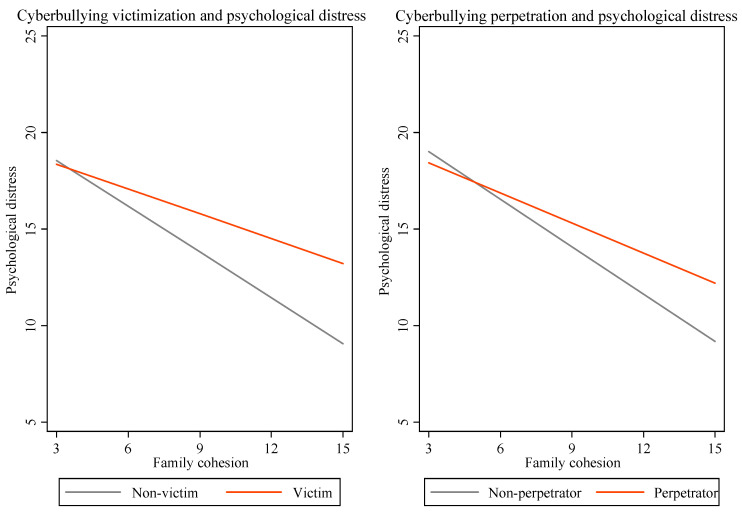
The moderating effects of family cohesion.

The correlations between cyberbullying involvement and psychological distress were analyzed using Ordinary Least Square (OLS) regressions. The unadjusted models of cyberbullying victimization and cyberbullying perpetration had adjusted R^2^ 0.064 and 0.012, respectively. When the family cohesion and school cohesion indicators were included, the adjusted R^2^ increased to 0.264 (victim model) and 0.247 (perpetrator model). The full cyberbullying victimization model’s explanation power, including all independent variables, control variables, and interactive variables, was 0.286, while the same parameter for the cyberbullying perpetration full model was 0.268. The associations between cyberbullying victimization and psychological distress were reported in Table 2, while the correlations between cyberbullying perpetration and psychological distress were presented in Table 3. The moderating effects of family cohesion on cyberbullying involvement’s impact on psychological distress were represented in Figure 1.

### 3.1. Cyberbullying Victimization and Psychological Distress

Being cyberbullied is significantly and positively correlated with psychological distress (Table 2). In the model that only included the cyberbullying victim, the victim had a 4.19 (*p* < 0.001) higher distress score than the non-victims. Both the family cohesion and school cohesion’s protective roles were identified when they were included in the model, even when the gender, grade, boarding school status, and academic performance variables were controlled. However, when the family cohesion and victim, school cohesion, and victim interactive variables were included, only the family cohesion and victim’s interaction was significant. This means that family cohesion did reduce the impact of being cyberbullied on psychological distress, while school cohesion did not, although both variables played significant protective roles in reducing psychological distress. Moreover, when the family cohesion, the school cohesion, the interactive variables, and the control variables were included (model 4), the effect of being a cyberbullying victim on psychological distress was not significant anymore.

### 3.2. Cyberbullying Perpetration and Psychological Distress

As shown in Table 3, being a cyberbullying perpetrator had a significantly higher degree of psychological distress as well, and this effect remained significant when the control variables and the two cohesion variables were included. However, it changed to non-significant when the interactive variables between being a perpetrator and cohesions were included. In the unadjusted model, the cyberbullying perpetrator had a 3.38 (*p* < 0.001) higher degree of distress than the non-perpetrators, but this effect reduced to 1.43 (*p* < 0.001) when the family cohesion and school cohesion were included and then increased to 1.51 (*p* < 0.001) when the controlled variables were included. Similar to the correlation patterns between cyberbullying victimization and distress, the cyberbullying perpetration’s impact on psychological distress became non-significant when we included the interactions.

### 3.3. The Moderating Effects of Family Cohesion

As revealed in Table 2 and Table 3, we included two interactions in the analysis, but only the family cohesion exhibited the moderating effects between the cyberbullying involvement and psychological distress. The moderating effects of family cohesion were demonstrated in Figure 1. Overall, family cohesion reduced both the being cyberbullied and cyberbully others’ effects on psychological distress, and this protective effect was higher for being bullied. In contrast, the school cohesion’s moderating effect was not supported in the analysis. Moreover, in both full models, including the control variables and the interactions, the correlations between cyberbullying involvement (both being a victim and being a perpetrator) and psychological distress were surmised. Further, the mitigating effect on psychological distress from family cohesion was more substantial for being a cyberbullying victim than being a cyberbullying perpetrator, as shown by the slopes in Figure 1.

Besides, boys had lower psychological distress than girls, and students in higher grades tended to have higher degrees of distress. With good academic performance, a student would have a lower degree of psychological distress.

## 4. Discussion

In this study, we employed a representative sample of children and adolescents from Jiangsu Province, China, to investigate the correlations between cyberbullying involvement and psychological distress, with an additional purpose to explore the moderating effects of family cohesion and school cohesion. This study can contribute to the current knowledge of cyberbullying in the following ways.

First, we found cyberbullying involvement, either being a victim or being a perpetrator, predicts a higher degree of psychological distress, and the effect of being a victim is higher when the moderation effects are not considered; thus, the research hypothesis one is partially supported. The self-reported cyberbullying perpetration of our study is 2.05%, while the victimization is 7.49%, much lower than the prevalence of a survey from Henan, Chongqing, and Zhejiang, which identified 31.4% (1170) students as victims, and 16.6% as perpetrators [31], while similar to another study’s victimization rate as 9.5% [32]. Consistent with most of the prior studies linking the cyberbullying experience with psychological problems, both being a victim and being a perpetrator have higher degrees or larger probability of developing psychological distress or other problems.

However, both the cyberbullying involvements’ impacts on psychological distress become non-significant when the family cohesion, school cohesion, and the interactions with cyberbullying are included. Family cohesion and school cohesion have played two roles. First, both of them are protective factors of psychological distress. Individual, peer relationships, family, school, and community factors, are potential protective factors against the involvement of bullying and cyberbullying, as well as their potential sociopsychology consequences [22,33]. There are at least three ways of keeping children and adolescents away from cyberbullying from the intervention and risk prevention perspective. First, keep them away from internet access [33], which is obviously unrealistic for generation Z, who grew up with the Internet, and this strategy’s adverse side effects can be much larger than the potential cyberbullying in general. The best option is to restrict the cyberbullying before it happens, while another option is to provide support for those involved in cyberbullying, and studies regarding the benefits of the interventions to increase support are needed in the future.

Our analysis focuses on discovering the potential intervention stages by finding the impact mechanism between cyberbullying involvement and psychological distress. This analysis demonstrates that only family cohesion mitigated (moderated) the impact of cyberbullying on psychological distress, while the school cohesion’s moderating effect is not significant, and thus hypothesis two is supported, while hypothesis three is rejected. Although the school climate is vital for bullying prevention [28], the family factors seem more important for psychological support for a cyberbullying involvement [22,34,35]. Compared with traditional bullying, the impact of cyberbullying may be more invisible since it does not cause immediate physical harm. Moreover, most schools at pre-college level have regulations on cellphone use and internet access during school time. Thus, the interventions for mitigating cyberbullying and the related psychological consequences should focus on the family. 

It should be noted that our study has at least two limitations. First, it only covered a representative sample from one province in China, and thus the conclusions cannot be extended to all Chinese children and adolescents, since China is such a big country with different cultures. Prior school bullying studies have indicated the geographical differences in bullying prevalence [26,28,36]. Second, the inevitable cross-sectional nature of this study cannot really generate causal relationships between cyberbullying involvement and the occurrence of psychological distress. Therefore, cyberbullying studies covering a representative sample of youth in China and longitudinal studies should be conducted to understand the causal mechanisms between cyberbullying involvement and social-psychological and behavioral problems. 

## 5. Conclusions

We investigated the correlations between cyberbullying involvement and psychological distress among Chinese children and adolescents using a representative sample from Jiangsu province in China, and aimed to examine the moderating effects of family cohesion and school cohesion. Overall, involvement in cyberbullying, either as a victim or perpetrator, was associated with a higher degree of psychological distress, and the effect of being bullied was higher. Both family cohesion and school cohesion played protective roles in reducing distress. Nevertheless, only family cohesion can reduce the impact of cyberbullying involvement on psychological distress (moderating effect), and this reduction was more remarkable for cyberbullied victims than cyberbullying perpetrators. Besides, students in higher grades had higher distress degrees, while the male students and those with good academic performance had lower degrees of psychological distress compared with their counterparts.

## Figures and Tables

**Table 1:** Descriptive analysis (N = 4978).

Variables	Frequency/Mean (SD)	Percent/Range
Dependent variable		
Psychological distress	11 (4.35)	5–25
Independent variables		
Cyberbullying victimization (yes = 1) ※	373	7.49
Cyberbullying perpetration (yes = 1) ※	102	2.05
Family cohesion ╪	12.82 (2.24)	3–15
School cohesion ╪	8.46 (1.41)	2–10
Control variables		
Gender (male = 1) ※	2561	51.45
Boarding (yes=1) ※	1189	23.89
Grade ╪	8.01 (2.00)	4–11
Academic performance ╪	3.40 (0.99)	1–5

Note: ※ frequency and percent were reported, ╪ mean (standard deviation) and range were reported.

**Table 2:** Cyberbullying victimization and psychological distress with moderating effects.

	Psychological Distress			
	Model 1	Model 2	Model 3	Model 4
Cyberbullying victim	4.19 *** (0.23)	2.38 *** (0.21)	2.36 *** (0.20)	−0.93 (1.21)
Family cohesion		−0.52 *** (0.03)	−0.51 *** (0.03)	−0.54 *** (0.03)
School cohesion		−0.83 *** (0.04)	−0.72 *** (0.04)	−0.72 *** (0.05)
Family cohesion interacting with cyberbullying victimization				
Family cohesion # non-victim				0.00
Family cohesion # Victim				0.24 ** (0.08)
School cohesion interacting with cyberbullying victimization				
School cohesion # non-victim				0.00
School cohesion # Victim				0.08 (0.15)
Gender (male)			0.79 *** (0.10)	−0.79 *** (0.10)
Grade			0.19 *** (0.03)	0.19 *** (0.03)
Boarding (yes)			0.24 (0.14)	0.25 (0.14)
Academic performance			−0.22 *** (0.06)	−0.21 *** (0.06)
Constant	10.68 *** (0.06)	24.55 *** (0.38)	23.59 *** (0.44)	23.95 *** (0.45)
Adjusted. R^2^	0.064	0.264	0.284	0.286

Note: # means interaction; Beta value reported; Standard errors in parentheses; ** *p* < 0.01, *** *p* < 0.001.

**Table 3:** Cyberbullying perpetration and psychological distress: the moderating effects.

	Psychological Distress			
	Model 1	Model 2	Model 3	Model 4
Cyberbullying perpetrator	3.38 *** (0.43)	1.43 *** (0.38)	1.51 *** (0.38)	0.61 (2.03)
Family cohesion		−0.56 *** (0.03)	−0.54 *** (0.03)	−0.55 *** (0.03)
School cohesion		−0.88 *** (0.04)	−0.77 *** (0.05)	−0.76 *** (0.05)
Family cohesion interacting with cyberbullying perpetration				
Family cohesion # non-perpetrator				0.00
Family cohesion # perpetrator				0.32 * (0.15)
School cohesion interacting with cyberbullying perpetration				
School cohesion # non-perpetrator				0.00
School cohesion # perpetrator				−0.35 (0.29)
Gender (male)			−0.80 *** (0.11)	−0.80 *** (0.11)
Grade			0.20 *** (0.03)	0.20 *** (0.03)
Boarding (yes)			0.22 (0.14)	0.22 (0.14)
Academic performance			−0.21 *** (0.06)	−0.21 *** (0.06)
Constant	10.93 *** (0.06)	25.54 *** (0.38)	24.49 *** (0.43)	24.56 *** (0.44)
Adjusted. R^2^	0.012	0.247	0.267	0.268

Note: # means interaction; Beta value reported; Standard errors in parentheses; * *p* < 0.05, *** *p* < 0.001.
